# Historical biogeography of the land snail *Cornu aspersum*: a new scenario inferred from haplotype distribution in the Western Mediterranean basin

**DOI:** 10.1186/1471-2148-10-18

**Published:** 2010-01-20

**Authors:** Annie Guiller, Luc Madec

**Affiliations:** 1 Laboratoire de Parasitologie Pharmaceutique (CNRS UMR 6553), Faculté des Sciences Pharmaceutiques et Biologiques, 35043 Rennes, France; 2 CNRS UMR 6553, Campus de Beaulieu, 35042 Rennes, France

## Abstract

**Background:**

Despite its key location between the rest of the continent and Europe, research on the phylogeography of north African species remains very limited compared to European and North American taxa. The Mediterranean land mollusc *Cornu aspersum *(= *Helix aspersa*) is part of the few species widely sampled in north Africa for biogeographical analysis. It then provides an excellent biological model to understand phylogeographical patterns across the Mediterranean basin, and to evaluate hypotheses of population differentiation. We investigated here the phylogeography of this land snail to reassess the evolutionary scenario we previously considered for explaining its scattered distribution in the western Mediterranean, and to help to resolve the question of the direction of its range expansion (from north Africa to Europe or *vice versa*). By analysing simultaneously individuals from 73 sites sampled in its putative native range, the present work provides the first broad-scale screening of mitochondrial variation (cyt *b *and 16S rRNA genes) of *C. aspersum*.

**Results:**

Phylogeographical structure mirrored previous patterns inferred from anatomy and nuclear data, since all haplotypes could be ascribed to a B (West) or a C (East) lineage. Alternative migration models tested confirmed that *C. aspersum *most likely spread from north Africa to Europe. In addition to Kabylia in Algeria, which would have been successively a centre of dispersal and a zone of secondary contacts, we identified an area in Galicia where genetically distinct west and east type populations would have regained contact.

**Conclusions:**

Vicariant and dispersal processes are reviewed and discussed in the light of signatures left in the geographical distribution of the genetic variation. In referring to Mediterranean taxa which show similar phylogeographical patterns, we proposed a parsimonious scenario to account for the "east-west" genetic splitting and the northward expansion of the western (B) clade which roughly involves (i) the dispersal of ancestral (eastern) types through Oligocene terranes in the Western Mediterranean (ii) the Tell Atlas orogenesis as gene flow barrier between future west and east populations, (iii) the impact of recurrent climatic fluctuations from mid-Pliocene to the last ice age, (iv) the loss of the eastern lineage during Pleistocene northwards expansion phases.

## Background

The aim of phylogeography is to explain the spatial distribution of genetic lineages within species and highlight the most influential historical factors explaining their distribution [1]. Hypotheses commonly advanced in phylogeography are inherent to dispersal and vicariant events, inferences about which being usually based on estimates of genetic diversity, divergence time and demographic history [2]. Inferences on demographic processes have especially become a central challenge in phylogeography and the recent development of analytical tools based on coalescent theory is very helpful for investigating the demographic history of populations. From information provided by phylogenetic trees and the signature in the pattern of DNA substitutions left in the case of historical change in population size, it is possible to identify the demographic process which has occurred and estimate related parameters [3,4] The wealth of literature dealing with phylogeography and demography in many invertebrate and vertebrate species emphasizes the importance of combining both lineage and population components to reveal biogeographical histories. Moreover, the comparison of phylogeographic structures across co-distributed taxa allows to find concordant partitions and to infer common historical factors of divergence and colonization. Then, in North America (for review see [5]) as in Europe [6], various species have been studied to determine the climatic and geological effects on phylogeographic patterns and population structures. More precisely in Europe, the Mediterranean basin characterized by an exceptional level of biodiversity and a complex geological history [7], has been the subject of intensive studies carrying out on genetic diversity and phylogeography. Nevertheless, most of these studies provide insights into the evolutionary history of southern European species. Despite its key location between the rest of the continent and Europe, only a few cases concern the north African territory. In addition, investigations including Algerian data sets are relatively rare, and main historical process influencing the differentiation is generally found in Quaternary climatic oscillations, meaning that few studies imply events which occurred over times much longer than the last glacial period.

**Figure 1:**
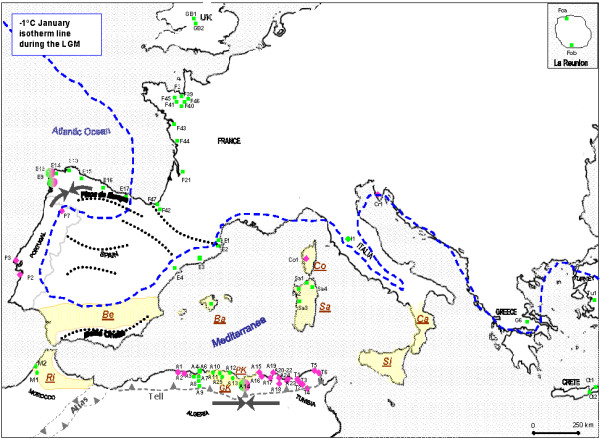
**Sampling locations of *C. aspersum *throughout the western Mediterranean basin**. Population numbers are those given in additional file 1. Sample site symbols and colours indicate the mtDNA lineage of the population: pink for east type, green for west type (including Z). Grey areas represent microplates drifted from Oligocene: **GK**: Great Kabylia, **PK**: Lesser Kabylia, **Ri**: Rif Cordillera, **Be**: Betic range, **Ba**: Balearic Islands, **Sa**: Sardinia, **Co**: Corsica, **Si**: Sicilia, **Ca**: Calabria. The dotted line throughout Europe shows the -1°C January isotherm line during the LGM [18]. Dotted curves in Iberia represent seven putative terrestrial refugia [69]. Putative geographical barriers (Moulouya River basin, Edough Peninsula, Atlas and Tell system) and contact zones (black arrows in opposite direction in Kabylia, Galicia and Italia) suggested in the present paper are also reported.

**Figure 2:**
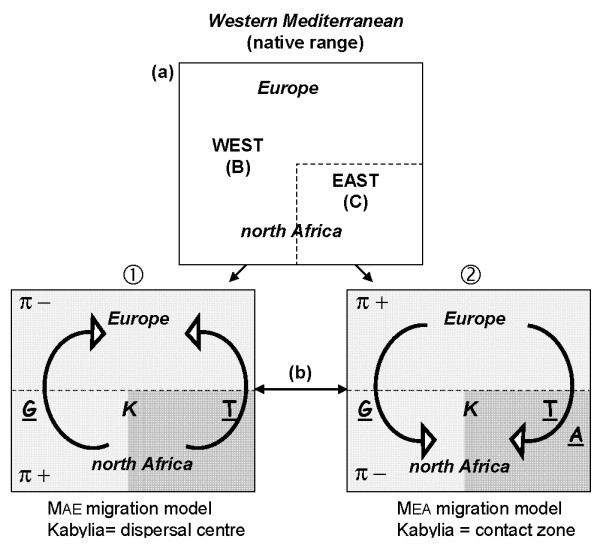
**Alternative biogeographical hypotheses for the current distribution of *C. aspersum *in Western Mediterranean and consequences in terms of spatial differentiation and genetic diversity**. (a) schematic representation of the West (B) and East (C) lineages defined in the native range of the species, (b) colonization routes following the two migration models tested (model 1 or MAE: migration from north Africa to Europe, model 2 or MEA: migration from Europe to north Africa) (K: Kabylia, **A**: Aegean route, **G**: Strait of Gibraltar, **T**: Tyrrhenian route, π: genetic diversity).

Amongst the few species widely sampled in north Africa (i.e. throughout the northern territory from western Morocco to eastern Tunisia) for studying historical processes influencing their current distribution, the land mollusc *Cornu aspersum* (= syn. *Helix aspersa*) provides an excellent biological model to understand phylogeographic patterns across north Africa and surrounding regions of the Western Mediterreanean basin, and evaluate hypotheses leading to population differentiation. This land snail, formerly known as *Helix aspersa *Müller, 1774, is originated from Mediterranean countries. It comprises a set of north African endemic forms and subspecies that were described at the beginning of the 20^th ^century on the basis of shell characteristics [8]. The most common one, *C. a*. *aspersum *(syn. *H. a. aspersa*), has become very abundant in all man-disturbed habitats in regions with a Mediterranean, temperate and even subtropical climate. To reconstruct the biogeographical history of this invasive form in the western Mediterranean, variation in spatial patterns of shell, genital and molecular characters was previously estimated by investigating more than hundred of populations representative of the *aspersum *range (western Mediterranean and European coastlines) (Fig 1). Almost all samples were examined for allozyme and morphometric variation [9-14] whereas only north African populations were analysed using part of the mitochondrial large subunit (16S rRNA) gene [15]. Whatever the set of populations and/or markers used, the combination of all different types of data leads to a clear pattern in geographical structure. Indeed, (i) two anatomically and biochemically divergent groups of populations (West *vs *East) between which the separation occurs in Kabylia (Algeria) is invariably observed throughout the north African coastal region; (ii) almost all European populations clustered with western north African ones, with smaller genetic distances than those between western and eastern north Africa (Fig 2), [9,10].

Two competing biogeographical scenarios based on opposite directions of dispersal were considered to explain the spatial pattern of variation in *C. aspersum *and especially to determine processes responsible for genetic isolation in the eastern part of north Africa (Fig 2) [10]. Both were based on Pliocene geological events and Quaternary cold periods, but colonization routes taken by the species involved migrations either from north Africa to Europe or from western Europe to north Africa, with likely secondary contacts in the Kabylian area. The doubtful direction of species expansion results from discrepancies of spatial structure between allozyme and partial mitochondrial data, and from an inconstant pattern of gene diversity. The previous scenario we proposed [9,14], implying a northwards progression of the species (model 1, Fig 2), fits well with published reports that place the origin of *C. aspersum *in north Africa [8]. However, mitochondrial gene diversity estimated in north Africa does not corroborate the northward colonization hypothesis [15]. The value of nucleotide diversity, twice as weak in the east (clade C) as in the west (clade B) north African mtDNA lineages, together with unresolved relationships between eastern populations, is rather consistent with southward dispersal either via Tyrrhenian/Aegean routes or the Strait of Gibraltar (model 2, Fig 2) [15]. Whilst in the first scenario we ascribed the lower diversity in east to the occurrence of transitory and prolonged bottlenecks inherent in successive migrations that populations experienced from Kabylia to Tunisia, this may also simply indicate a more recent divergence within the eastern region.

These two hypotheses were evaluated here by studying the phylogeography and the demographic history of *C. aspersum *over all European and African populations sampled. Since parameters relevant to fluctuating population size on the evolutionary time scale were never taken into account in previous phylogeographical studies of the species, we hoped that their estimation would help to shed more light upon the scenario we previously considered to account for the scattered distribution of *C. aspersum *[9,10]. The aim of this work is to assess the geographic patterns of mtDNA diversity in order (i) to improve our understanding of the species range expansion and infer the dispersal direction, (ii) to elucidate the role of the Kabylian area suspected until now to be a suture zone in Algeria. Without ruling out the fact that demographic processes and selective sweeps can lead to convergent genetic signatures when only one genetic marker is analysed, we hope that demographic estimates will allow to reinforce one of the hypotheses relating to the sense of dispersion in western Mediterranean, as well as that concerning the likely contact zone in Kabylia. Recent demographic expansion in the western lineage, combined with low nucleotide diversity, should favour a south-north dispersal, whilst a recent colonization from northern locations to the south should indicate demographic stability and high molecular diversity indices within the western lineage (Fig 2).

## Results

### Sequence variability

A 557 bp sequence of the mtDNA cytochrome *b *(cyt *b*) was obtained for 114 *C. aspersum *individuals. To reduce the homoplasic effect of transitions on tree reconstruction, especially at higher levels of divergence, we excluded the highly variable third codon position from the analysis. A total of 89 mutations were then scored. The fragment contained 75 variable sites leading to a nucleotide diversity of 0.036 ± 0.019 and defining 62 different haplotypes of which 39 were unique. As a result, the overall haplotype diversity was high (0.98 ± 0.01) (Table 1). Sequences from the 16S rRNA were obtained for 128 *C. aspersum *individuals. They had a length of 377 bp. The total number of mutations was 123. The nucleotide diversity was high (0.057 ± 0.028) covering 89 polymorphic sites of which 61 were parsimony informative. A total of 75 haplotypes (excluding sites with gaps and missing data) were identified, producing a high overall haplotypic diversity (0.98 ± 0.01) since 56 of them were unique (Table 1).

**Table 1:** Genetic diversity estimates and results of demographic tests for cyt *b *and 16S genes in main geographical subdivisions of *C. aspersum*: B1, B2 for cyt *b *and Ba, Bb for 16S are subgroups of the B lineage, C1 and C2 are subgroups of the C lineage; Z includes intermediate zone sequences.

Gene	**Geo. subdiv**.	Sample size	*h*	*Diversity index*		*Demographic tests*	
				*H *± sd	θπ ± sd	*Fs*	*R*_2_
**Cyt *b***	**B#**	68	31	0.95 ± 0.01	9.34 ± 4.34	-6.38*	0.083
	**C**	46	31	0.98 ± 0.01	8.52 ± 4.01	-13.43 ***	0.093
	**B1**	15	8	0.90 ± 0.05	2.23 ± 1.30	-2.69 *	0.103 *
	**B2**	40	16	0.89 ± 0.03	3.13 ± 1.84	-5.89 **	0.060 *
	**C1**	10	7	0.91 ± 0.08	6.29 ± 3.69	-0.2	0.182
	**C2**	36	24	0.97 ± 0.02	7.18 ± 3.83	-9.43 **	0.095
	**Z**	13	7	0.89 ± 0.06	12.42 ± 6.74	3.02	0.189
	**all**	114	62	0.98 ± 0.01	13.8 ± 6.23	-23.63 ***	0.087
							
**16S**	**B#**	81	41	0.95 ± 0.01	15.27 ± 6.83	-6.58	0.096
	**C**	47	34	0.98 ± 0.01	13.20 ± 6.04	-11.09**	0.078
	**Ba**	23	15	0.95 ± 0.03	4.18 ± 2.4	-6.38 ***	0.069 ***
	**Bb**	49	21	0.88 ± 0.03	3.70 ± 2.11	-9.17 ***	0.059 *
	**Z**	9	5	0.81 ± 0.12	15.64 ± 8.76	4.48	0.165
	**all**	128	74	0.98 ± 0.01	21.56 ± 9.57	-22.81 ***	0.090

*h*: number of haplotypes; *H*: gene diversity; θπ: nucleotide diversity; sd: standard deviation; *Fs: *Fu's F statistic; *R*_2_: Ramos-Onsins & Rozas' statistic; significance levels for *Fs *and *R*_2_: *<0.05; **<0.01; ***<0.001.

**# **including Z sequences.

### Phylogenetic and network relationships among haplotypes

The left-skewness test for the cyt *b *gene indicated that the observed tree length distribution was significantly more skewed than expected from random expectation (g1 = -0.193, *p *< 0.01). The g1-value obtained for 16S rRNA (g1 = -0.109, *p *< 0.01) was also indicative of a highly significant structure. The hypothesis of homogeneous rate within alignment of 16S and cyt *b *genes respectively was not rejected by the molecular clock tests. The likelihoods with no molecular clock enforced were not significantly better than those with enforced molecular clock (cyt *b*: 2ΔlogL = 15.8, d.f. = 60, *p *= 1; 16S, 2ΔlogL = 64.3, d.f. = 72, *p *= 0.73). For both genes, the RRT detected no significant heterogeneity between the defined lineages. Calculation of divergence time from phylogenies was then justified.

**Figure 3:**
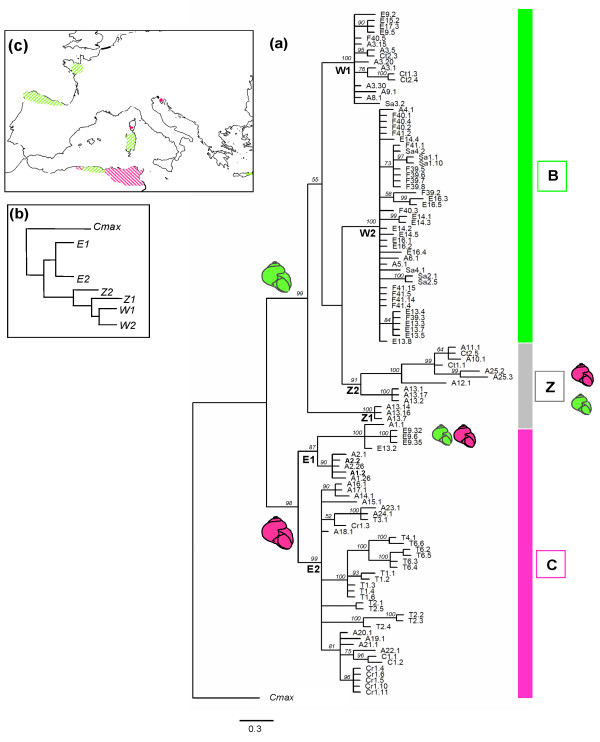
**Phylogenetic relationships among cyt *b *sequences**. (a) Fifty percent majority-rule consensus phylogram from the BI analysis. Branches without posterior probability values (values in italics) are supported by less than 50% of the sampled trees. Sequence labels are abbreviated as in additional file 1. Main subdivisions are indicated on the right side of the tree (B and C lineages), and inside each subdivision, the respective clades are presented. Schematic colored shells indicate morphometric (shell and distal genitalia) type of each subdivision (green for B or west type, pink for C or east type). (b) Schematic representation of ML topology. (c) Schematic geographic location of west (B, green) *vs *east (C, pink) haplotypes.

The topology of the cyt *b *trees obtained by ML and BI approaches revealed two strongly supported monophyletic groups B and C (Fig 3). In one group (C clade), sequences of all eastern populations of north Africa (from east Kakylia to Tunisia) clustered with Corsican and Croatian samples defining the C2 lineage. Neither haplotypes from the Croatian localities nor those from the Tunisian ones turned out to form monophyletic sub-groups in accordance with expectations of geographical positions. Sequences of both western Algerian samples A_1 _and A_2 _were also included in the C group but form a paraphyletic lineage (C1) in which four sequences from Galicia in Spain were also included. The topology of the sister group (B clade) was shallow and there were few significant genealogical branches of samples corresponding to sampling locality. Except individuals originating mostly from the Kabylian zone in Algeria (A_10-13 _and A_25_; lineage Z1 and Z2) and most French ones that were monophyletic (lineage B2), individuals from western north Africa (from west Kabylia to Morocco), Spain, Crete and Sardinia were scattered throughout the B group.

**Figure 4:**
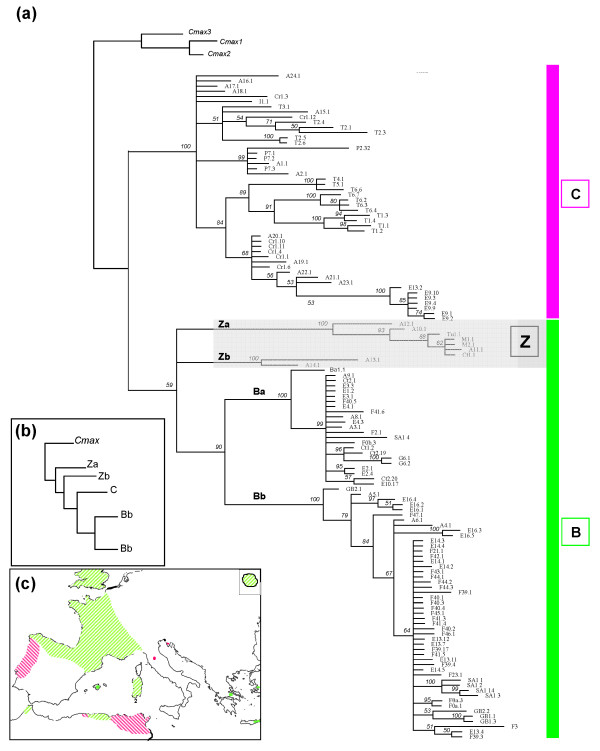
**Phylogenetic relationships among 16S RNA sequences**. (a) Fifty-percent majority-rule consensus phylogram from the BI analysis (see legend of Fig 3 for details). (b) Schematic representation of ML topology. (c) Schematic geographic location of west (B, green) *vs *east (C, pink) haplotypes.

The 16S RNA tree's topologies yielded identical grouping of individuals as Cyt *b*, with the emergence of both B and C clades (Fig 4). ML and BI topologies were different mainly in the position of two lineages which clustered nine individuals from populations in Kabylia, Morocco, Crete and Turkey. These two clades, which were the most basal on the ML tree (tree not shown), formed two sister clades within the B lineage on the BI tree. Sequences from sampling locations not analysed for cyt *b *were scattered throughout the trees. Italian and Portuguese individuals were included in the C group whereas sequences from Greece, England and newly analysed French populations clustered within the B lineage.

**Figure 5:**
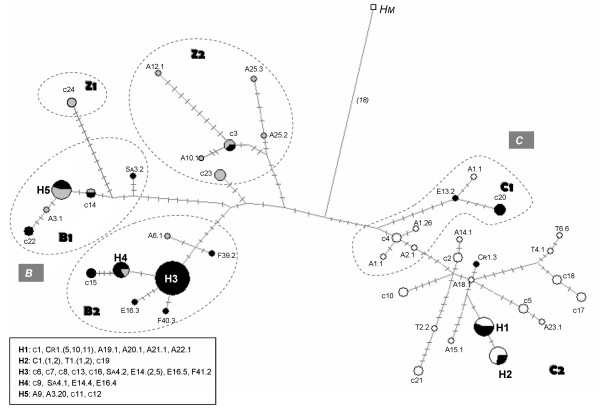
**Median-joining network for the cyt *b *mtDNA haplotypes of *C. aspersum***. *H *haplotypes define ancestral types resulting from the star contraction analysis. Inferred median vectors are switched off for clarity. Each circle represents a haplotype, and circle size is proportional to haplotype frequency. Colours indicate the subdivisions inside haplogroups: white for east (C) and grey for west (B) north African types as previously defined [15], black for west European types. Dashed lines delineate haplogroups defined from cyt *b *phylograms. Branch lengths are approximately equal to inferred mutational steps (m) (short lines perpendicular to branches for *m*<10, numbers in brackets for *m *≥ 10). Haplotype codes according to those in additional file 1.

For both genes, the haplotype network corroborated the existence of two main phylogroups, B and C (Fig 5, network not shown for 16S). Geographical discontinuities within both haplogroups were also confirmed with the Croatian and Corsican haplotypes linked to the Numidian (east Algeria) ones in C, and the Sardinian and Crete haplotypes widespread through the B lineage. Interestingly on the cyt *b *network is the position of both B and C haplogroups each side of *C. a. maxima *haplotype. For the C haplogroup, Tunisian, eastern Algerian and all C2 haplotypes would emerge from individuals originating from western Algeria (A_1 _and A_2 _populations). Moreover, Spanish haplotypes from E_9 _and E_13 _should also originate from this western Algerian area. For the B haplogroup, both distinct B1 and B2 lineages seem to have originated and diverged independently from the Kabylian area in Algeria. In these two B sub-clades, the network was also indicative of a more recent population expansion in which several localizated lineages were connected by short branches to the most common haplotype that occurred in French or Spanish populations. A high level of mitochondrial diversity both within and among lineages clearly appears from the examination of both the 16S and cyt *b *networks. The nucleotide diversity θ_π _was higher in B than in C lineage (Table 1). However, this variability became much lower in B than in C when the Z sequences are removed from the B clade (θπ_cyt*b *_= 6.8 ± 3.6; θπ_16S _= 11.5 ± 5.8; θπ_(cyt*b*+16*S*) _= 12.9 ± 6.7). As suggested by the short branches in B subclades, the magnitude of change was lower in B1 and B2 for cyt *b *than in Ba and Bb for 16S.

### Pattern of migration

For both genes analysed separately, results of AIC values comparing four migration models support an asymmetric model of migration from north African to European populations of the B lineage (Fig 2). The model 1 (MAE) had an AIC value of 6.0 and 5.9 for cyt *b *and 16S respectively, whilst values of the MEA (model 2) were 730.3 for cyt *b *and 14 92.1 for 16S.

### Demographic analyses

**Figure 6:**
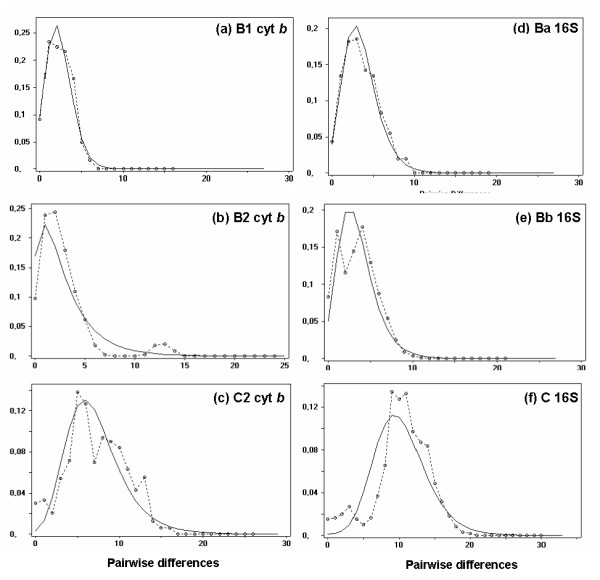
**Mismatch distributions found in subdivisions (a) B1, (b) B2 and (c) C2 for cyt *b *sequences, (d) Ba, (e) Bb and (f) C for 16S RNA sequences**. The continuous and interrupted (connecting circles) lines indicate the expected and observed distributions of pairwise differences obtained by fitting a model of sudden population expansion [89].

We used haplogroups based on both genes to estimate demographic parameters to test for demographic events. Regarding the cyt *b *gene, a model of constant size could not be rejected for most haplogroups, except for clades B1 and B2 (both Fu's test and *R*_2_; Table 1). Fu's test only was significant for clade C2. These results were confirmed by the observed mismatch distributions which closely matched those expected under a model of sudden expansion for these three haplogroups (Fig 6a, b, c). Fu's and/or *R*_2 _tests performed with 16S sequences of the B clade (in the whole population and in Ba and Bb haplogroups) also rejected the null hypothesis of constant size (Table 1). As suggested by the bimodal pattern of mismatch distribution in B (results not showed), the presence of two subgroups Ba and Bb may explain the non-significant *R*_2 _in group B. By contrast, the unimodal distribution of pairwise differences among haplotypes in Ba and Bb haplogroups perfectly fitted the distribution predicted by the sudden expansion model (Fig 6d, e). Populations of the C clade may also have experienced demographic expansion (Fig 6f) (Table 1).

**Figure 7:**
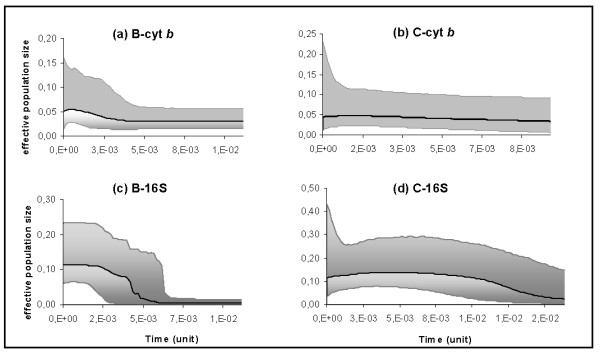
**Bayesian skyline plots showing the historical demographic trends for both B and C lineages detected for cyt *b *(a and b) and 16S RNA (c and d) sequences**. Along the *y*-axis is the expressed population size estimated in units of *N*_*e*_μ (*N*_*e*_: effective population size, *μ*: mutation rate per haplotype per generation). Solid lines are median estimates whereas shaded areas represent confidence intervals.

Historical demographic reconstructions (BSPs) of the B and C clades are shown in fig. 6. According to cyt *b *plots, after a phase of constant population size, the B haplogroup appeared to have experienced demographic expansions, one around 5.3 × 10^-3 ^(between 0.53 and 0.13 Myr with 0.5% and 2% respectively) and one more discrete around 0.37 × 10^-3 ^(between 37 000 and 9 300 years with 0.5% and 2% respectively) time units (Fig 7a). A somewhat similar trend was observed for the 16S gene, for which a faster population growth would have occurred at around 6.4 × 10^-3 ^time units (between 0.64 and 0.16 Myr with 0.5% and 2% respectively) (Fig 7c). For the C lineage, both cyt *b *and 16S plots revealed a prolonged phase of demographic stability followed by a recent expansion, starting at around 1.5 × 10^-3 ^for cyt *b *(between 0.15 Myr and 38 000 years with 0.5% and 2% respectively) and 2 × 10^-3 ^time units (between 0.2 Myr and 50 000 years with 0.5% and 2% respectively) for 16S (Fig 7b, d). Estimates of TMRCA (and 95% HPD) based on the coalescent theory showed congruent values for 16S and cyt *b *genes (Table 2). According to fossil records and previous estimates based upon allozyme data, likely time values would correspond to divergence rates ranging from 0.5 to 2% per Myr. Assuming this rate divergence range, the most recent common ancestor of all *C. aspersum *would have lived between 6.1 (4.8-7.6) and 1.1 (0.7-1.5) Myr ago. After excluding the basal Z sequences on the BI tree for 16S, the split into the B and the C clades would have occurred between 4.2 (3.5-5.1) and 1.1 (0.9-1.3) Myr ago. The split between B subclades would have taken place around 3.2 (2.3-4.3) and 0.8 (0.5-1.1) Myr ago, while the most recent common ancestor of all C individuals would have lived between 2.6 (1.9-3.3) and 0.6 (0.4-0.9) Myr ago.

**Table 2:** Estimates of TMCRA (and 95% Highest Posterior Density) for both cyt *b *and 16S genes overall *C. aspersum *sequences and of the B and C lineages.

		cyt *b*						16S			
node											
divergence rate (%)		0.03	0.5	1	2	5		0.5	1	2	10
	*time unit*	% per Myr					*time unit*	% per Myr			
***C. aspersum***	*0.043*	72.2	4.3	2.2	1.1	0.4	*0.061*	6.1	3.1	1.5	0.3
**95% HPD lower**	*0.028*	46.6	2.8	1.4	0.7	0.3	*0.048*	4.8	2.4	1.2	0.24
**95% HPD upper**	*0.059*	97.6	5.9	2.9	1.5	0.6	*0.075*	7.6	3.7	1.9	0.38
											
B*	*0.031*	52.2	3.1	1.6	0.8	0.3	*0.032*	3.2	1.6	0.8	0.16
**95% HPD lower**	*0.020*	32.9	2	1	0.5	0.2	*0.023*	2.3	1.2	0.6	0.11
**95% HPD upper**	*0.044*	72.6	4.4	2.2	1.1	0.4	*0.043*	4.3	2.2	1.1	0.21
											
**C**	*0.024*	39.9	2.4	1.2	0.6	0.2	*0.026*	2.6	1.3	0.6	0.13
**95% HPD lower**	*0.015*	25	1.5	0.8	0.4	0.1	*0.019*	1.9	1.0	0.5	0.1
**95% HPD upper**	*0.034*	56.7	3.4	1.7	0.9	0.3	*0.033*	3.3	1.7	0.8	0.17

A range of substitution rates in percentage per Myr was tested, from 0.03 to 5 for cyt *b*, from 0.5 to 10 for 16S. * including Z for cyt *b *estimates

## Discussion

Our results based on 16S and cyt *b *mtDNA variation showed phylogenetic relationships among *C. aspersum *haplotypes consistent with inferences based on anatomy, nuclear (allozyme) and mitochondrial data previously analysed [9,11,12,15]. The split into two haplogroups is supported since haplotypes of populations newly investigated compared to the previous mtDNA study [15] scattered either in the B or in the C lineage without really forming a new one. In a wider sense of the nomenclature inferred from these previous works, these B and C lineages were named "west" and "east" respectively, although the origin of some samples is not always in accordance with this spatial identification. Indeed, both lineages can include haplotypes originating from geographically distant populations. These discrepancies between genetic and geography are suggestive of a relatively intricate phylogeographical history of *C. aspersum*. As we previously showed, the genetic structuring of populations does not seem to be simply a function of dispersal [10]. Beyond vicariance involving Tertiary tectonic events and Pleistocene glaciations, migration due to human actions needs to be mentioned to account for the current distribution of the species outside north Africa. However, the relative role of each process seems to vary greatly from one region to another. Before assessing the contribution of each of the different processes and refining the evolutionary scenario for the emergence of the two distinct lineages, we will recapitulate the main results by focusing particularly on contrasting zones of the distribution area that appear to be major historical entities.

### The key areas detected

The tricky question of the dispersal scenario of the species can be solved by the genetic information provided by the northern part of the species range and preliminary outcomes of northward *versus *southward migration models tested. Within this wide area represented by western sublineages (i.e. B2 for cyt *b*, and Bb for 16S), four points can argue in favour of a northward movement: the better support of an asymmetric dispersal model towards a north Africa to Europe migration (model 1, Fig 2), the very low nucleotide diversity obtained, the star-like structure of the haplotypes network, the presence of Algerian individuals scattered in all subclades knowing that such splitting concerns exclusively Algerian samples. Although not specific to these sublineages, recent demographic expansions registered in B2 and Bb strengthen this dispersal direction that could have possibly started from Algiers area (represented by A_4_, A_5_, A_6 _populations). Expansion time estimates are purely speculative since no calibration point is available. However, values inferred from divergence rates fitting with previous allozyme estimates (see below), i.e. from 1 to 5% per Myr, would suggest that expansions highlighted by historical demographic reconstructions would have occurred twice. The first would have happened during one of the Pleistocene interglacial periods (i.e. from around 700 000 years ago), and the second at mid-Holocene after the maximum extent of the ice sheets during the last glaciation (between 37 000 and 7 400 years ago).

One area in Algeria seems to have played a central role in the dispersal of the species. Both genes showed that the Kabylies (represented by samples A_10_-A_14 _and A_25_) would have constituted a significant centre for dispersal and differentiation (model 1, Fig 2). However, phylogenies display an unstable position of Kabylian sequences. They occupy a basal position within the western lineage except on the ML 16S tree where they form a sister group of the remaining individuals of *C. aspersum*. The population of Djemila (A_14_) is an exception since the cyt *b *haplotype of this sample lies within an eastern subclade that also includes haplotypes of neighbouring populations (A_15_, A_16_, A_17_). Therefore, the Kabylies could possibly be the ancestral range of either *C. aspersum *or of the western lineage only. A difficulty in distinguishing between these two hypotheses stems from genetic exchanges that could have then occurred between western and eastern populations, leading to disruption in genetic structuring (model 2, Fig 2). The pattern of allele distribution at 13 polymorphic allozyme loci in intermediate Algerian populations ranging from Azzefoun (A_12_, Great Kaylia) to El Hedaick (A_16_, Skikda) reflects the possibility that peripheral populations of both East and West North-Africa would have regained contact after having been isolated. As previously suspected with the occurrence of eastern typical allozymes found in Djemila [16], introgressive hybridization between snails of the mixed population could also explain the presence of one C (east) typical cyt *b *haplotype in Djemila. The derived position of this haplotype diverging from A_16 _would moreover support the assumption of horizontal transfer of the mitochondrial genome through hybridization. One might suppose that both C (east) and B (west) haplotypes coexist in that population but quite obviously, more individuals from Djemila and surrounding sites need to be analysed to refine the gene diversity and reinforce the gene exchanges assumption between lineages in contact. Evidence in favour of this contact model is the west-east clinal decrease in a pair of genital organ lengths, i.e. the bursa tract diverticulum and the flagellum [14]. Suspected hybridizing populations and especially those from Djemila (A_14_), El Ancer (A_15_) and El Hedaick (A_16_) have medium-sized organs compared with eastern and western ones. Therefore, whether the Kabylies is a dispersal centre or not, it is likely a zone where genetically distinct individuals from east *versus *west met and mated, resulting in at least some offspring of mixed ancestry. It is worth noting that other individuals sampled outside the Kabylies join this putative dispersal centre, especially snails from Morocco, Crete and Turkey.

The C clade equivalent to the eastern lineage we previously defined from anatomy, shell and molecular features covers here a larger geographical range. In addition to original samples from eastern north Africa, western Algeria (A_1 _and A_2_) and Italy [9-11,15], samples more distant geographically join this clade. These are isolated specimens from Corsica, Croatia (Istria) and the western coast of Iberia (Cape Finisterre and Muxia in Galicia, coastal sites in Portugal). Although there is no clear evidence to explain this grouping, we can nevertheless point out that all populations of this lineage are outside the zone defined by the -1°C January isotherm during the last glacial maximum (LGM), i.e. about 18 000 BP [17,18]. Contrary to populations of the B lineage that might have expanded northwards after glacial episodes from either southern differentiation centres or after introductions, populations of the C clade would have been limited in their post-last glacial maximum dispersal.

### The biogeographical scenario reconsidered: new insights into haplotype distribution in north Africa and Europe

*Cornu aspersum *is a typically anthropochorous species which is nowadays widespread throughout the world in many zones with climates differing from the original Mediterranean one. Its presence is reported on the American continents, as well as in Australia and in Asia. Therefore, the first explanation for resemblances between populations located on either side of the Mediterranean could be passive transport due to human activities. Transfers might have started as nearly as the Neolithic revolution (around 8 500 BP; [9,12]) and nowadays they continue occurring in giving rise in some cases to catastrophic destruction of habitats. Such man-made dispersal is however insufficient to explain the genetic pattern of variation obtained. Signatures left in the geographical distribution and the genetic diversity of extant populations indicated differences in spatial patterning between north Africa and Europe that could not be interpreted as a result of human-assisted dispersal alone. While a real pattern of regional differentiation exists for African samples [12], a lack of genetic structure due to a great admixture of populations is detected in northern parts of the species range [10]. Hence, European individuals of the same population might originate from distinct native populations more or less differentiated genetically. Different waves of dispersion could explain marked genetic divergence estimates among the homogeneous groups of populations we defined using spatial contiguity constraint (see [10] for details). For the B lineage, northern and eastern Spanish areas like French ones should have been twice colonized by recurrent individuals coming from native populations of the same region. By contrast, snails from south and west Iberia that exhibit genetic similarities with north African individuals would have originated from more highly differentiated sources, probably Europe and north Africa. The Galician populations of Cape Finisterre (E_9_) and Muxia (E_13_) mirror such findings with an extremely frequently recorded level of mitochondrial diversity. The co-occurrence within a given population of both B and C specific haplotypes contributes to extreme divergences (for cytb: θ_π __(E9) _= 13.8 and θ_π __(E13) _= 6.9; for 16S: θ_π __(E13) _= 11.8 and θ_π __(E9) _= 0.5 with only eastern types present in E_9_) that consequently leads to discord between gene and population trees. This intermixing of distinct haplotypes is questionable since it occurs only in one part of the species range which moreover is peripheral and not expected to be the differentiation centre of *C. aspersum*. The overall differentiation between these sympatric divergent haplotypes involves two likely sources of variation: (i) divergence which already existed within the common ancestral populations; (ii) divergent haplotypes originating from previously isolated lineages [19]. These components of variation imply underlying debatable mechanisms such as incomplete lineage sorting of ancestral polymorphisms due to recently divergent populations or recent migration events between allopatric populations [20,21]. Incomplete lineage sorting from an ancestral polymorphic gene pool could possibly be the cause of the mitochondrial polyphyly detected in E_9 _and E_13_. The high gene diversity observed overall *C. aspersum *sequences compared to the number of individuals analysed prevents reliable differentiation between shared (ancestral) and specific (derived) haplotypes. However, a distinction could be proposed using their position on networks. Then, eastern haplotypes for both genes would be specific to E_9 _and E_13 _whereas western mitotypes would be either specific to each population or shared by other samples more or less distant geographically (Iberian and French samples). For East types only, the absence of ancestral mitotype could correspond to the "allotypy" pattern of the intermediate polyphyly progression described by Omland *et al*. [22] and documented by other studies [23-25]. Intermixing of Iberian and East North-African haplotypes should then indicate that they would not be completely sorted. In that case, nuclear genes having about four times the effective population size of mitochondria, would however fail to locate populations which are reciprocally monophyletic [26]. Although E_9 _and E_13 _are clearly distinct genetically from most other Iberian samples belonging to the same B clade, nuclear variation is effectively consistent with monophyly of both populations with no intermixing of typical alleles of B (west) *versus *C (east) lineages. Allozyme data indicated few common alleles shared only with western north African populations, especially M_1_, M_2_, A_1 _and A_2 _[9], and assignment tests performed on microsatellite genotypes clustered almost all individuals of both samples (92 of 95 individuals analysed) within a single cluster rather than distinct ones [27]. Other arguments such (i) the occurrence of close western types of both Iberian populations in very remote geographical regions, (ii) the great genetic divergence estimated between the aforementioned intermixed B and C haplotypes, (iii) the geographical location of populations having non-monophyletic haplotypes, tend to argue against this hypothesis of incomplete sorting in promoting secondary contacts between previously isolated lineages. The pattern of morphometric distinctiveness between western and eastern typical populations also supports such hypothesis of migration between divergent populations. Only explanations based on secondary contacts between snails of the B and C lineages could clarify the morphometric heterogeneity in space and time registered in Galician populations, in which adult snails exhibit B or C shell and genital features according to sampling dates (1991 and 2004 for E_9_, 2004 for E_13_) and location. Comparison with the morphometric profile of snails originating from populations of the suspected contact zone in Kabylia may provide insights into the process responsible for the variation observed in Galicia. As mentioned above, snails from Djemila (A_14_), which can be considered as the most representative of the intermediate zone [16], have genitalia of an intermediate size, which is about average for the species [13,28]. Such an intermediate pattern of covariation between shell and genital organs should imply mechanism leading to an eventual precopulatory isolation between snails from the eastern and western lineages. More than a simple coexistence of two distinct morphotypes within a population, the intermediate size of diverticulum of snails from Cape Finisterre could reflect the evolution of premating reproductive isolation among lineages recently in contact...The contradictory genetic signature of mitochondrial *versus *nuclear loci may support this scenario in which horizontal transfer of distinct haplotypes would occur through introgressive hybridization between both lineages where reproductive barriers are incomplete. The mitotypes characterizing southern and western populations of the peninsula strengthen the putative contact zone inferred in that area since Portuguese samples (P_2_, P_3_, P_7_) showed C types only, whereas northern Iberian populations (E_10_, E_14_-_17_) have B types. An extensive sampling of this western part of Iberia is however imperative to corroborate this conclusion.

Inferring secondary contacts between genetically distinct west (B) and east (C) populations in Kabylia as in Galicia assumes that originally the species has undergone a strong genetic splitting. Time estimates based on present mitochondrial results (between 4 and 1 Myr) do not refute previous allozyme assessments (Nei's genetic distance between west and east subdivisions is between 0.25 and 0.18, leading to time estimates ranging from 3.1 to 1.8 Myr; [9,29]), and are both consistent with mid-Pliocene/Pleistocene break-up events. Except for global climate changes of the past 4 Myr including the end of the early warm period (5-3 Myr) and significant intensification of northern hemisphere glaciation around 2.75 Myr ago [30-32], there is no clear evidence of a past biogeographic event at the time of the shift which occurred in *C. aspersum*. However, in the expectation of an investigation that would compare the phylogeographic structures of Mediterranean taxa showing similar geographic and genetic splits (work in progress), we already offer a non-exhaustive synopsis of biogeographical studies of north African populations to infer historical factors that could explain the deep genetic break observed in Algeria ([18,33-62]; Table 3). The oldest event involved in the differentiation process relates to the tectonic evolution of the western Mediterranean region [31,32]. Several plant and animal taxa showing genetic shift geographically consistent with the fragmentation of those microplates would have then undergone differentiation through such a vicariant event [33-36,38]. Whether or not due to the plate tectonics of the region, the Tell Atlas and Atlas mountains uplifted between 13 and 11 Myr ago [63] from which two areas of endemism have been delimited in north Africa [64], the end of the Messinian salinity crisis leading to the opening of the Strait of Gibraltar 5.3 Myr ago, and the emergence of continental islands formed by successive marine floodings could have formed major significant biogeographical barriers in the westernmost area of the basin (Talbe 3). As for other taxa such as the newt *P. nebulosus *[43], the salamander *Salamandra algira *[49] and the land snail *Turodella sulcata *ss *versus Turodella *sp [50,51], the formation of the Moulouya River basin in Morocco and the fossil island called the "Edough Peninsula" in eastern Algeria (around 4.2 Myr ago), could effectively be responsible for genetic isolation of the species. Based on similarities in estimated divergence time (around 2 Myr) and spatial barrier (the Kabylies) between distinct lineages, it is tempting to think that Pliocene climatic fluctuations would have also contributed to the east-west subdivision of *Crocidura russula *in north Africa [55]. As we suggested previously [9], Pleistocene climatic changes involving the formation of glacial refuges would have afterward strengthened the split between both B and C lineages. Of north African refuges described in the past, two are located in the west and are separated by the Atlas Mountains in Morocco, while two others are in the Algerian-Tunisian Tell area [65,66]. However, the climatic fluctuations occurring in Europe during the Pleistocene would not have greatly affected the northern part of Africa (Frizon de Lamotte, comm. pers.). In spite of a large proximity between Europe and Africa, the glacial episode would really have had only minor effects on flora and fauna in north Africa [67]. Consequently, a scenario involving successive retreats and advances of snails into and from matorral formations described as potential refuges in the Kabylies and Tunisia during the last pleniglacial [9] must be regarded as tentative. The vegetation map reconstruction presented recently by Ray and Adams [68] indicates three main vegetation types at the LGM in north Africa: (i) a tropical woodland lying between the Algerian coastline and the Tell, (ii) a tropical semi-desert spreading from east north Africa (Tunisia) towards the southwestern Moroccan coast and corresponding to the Atlas domains, (ii) a tropical grassland south of the Rif and corresponding to the Moroccan Meseta. This vegetational pattern, showing a clear shift between deciduous woody vegetation in west and sparse (rocky) vegetation in east, fits better the west-east pattern of genetic variation with Kabylia at the intersection. Ecological constraint due to climatic changes would have then reinforced the B-C split occurred long before.

**Table 3:** Mediterranean taxa as examples of phylogenetic splits in north Africa (NA) (MSC: Messinian Salinity Crisis; ?: no available data).

Events	Divergence Time (Myr)	Taxa	Lineages	Reference
Tectonics (microplates)	Oligocene-Miocene	the cork oak *Quercus suber*	2 sub-lineages in Morocco *vs *1 in Tunisian	[33]
Tectonics (microplates	Oligocene-Miocene	the pine *Pinus pinaster*	Morocco *vs *Tunisian lineages	[34]
Tectonics (microplates) Geographical barrier: Moulouya River basin	9.5-5.6 1.8	the frogs *Discoglossus pictus-auritus*/*scovazzi*	2 isolated lineages in contact in NA: *D. pictus-auritus *in Algeria-Tunisa *vs D. scovazzi *in Morocco	[35,36]
?	(15-10)	the scorpion *Buthus occitanus*	Atlas (Morocco) *vs *Tell-Atlas (Tunisia) lineages	[37]
Tectonics (microplates)	?	the frog *Hyla meridionalis*	Morocco *vs *Tunisian lineages	[38]
Geographical barrier: Atlas Mountains in Morocco	13 - 11	the fresh turtle *Mauremys leprosa*	2 clades north *vs *south mountains	[39]
Geographical barrier: Atlas and Tell systems	13 - 11	the lizards *Lacerta pater/L. tangitana*	2 species west *vs *east mountains	[40]
Mid-Miocene split -end of MSC Marine transgression (Edough peninsula)	14-5.3 4.2	the newts *Pleurodeles waltl P.nebulosus P. poireti*	Differentiation *P.waltl/*ancestor *P.nebulosus-P. poireti * Differentiation *P.waltl/*ancestor *P.nebulosus-P. poireti*	[41-43]
Geographical barrier: Atlas uplift	8.5-9.4	the Agamid lizard *Agama impalearis*	2 lineages in Morocco: NW *vs *SE Atlas	[44]
MSC ?	6-3.5	the snake *Malpolon monspessulanus*	*M.m*. *monspessulanus *from Morocco/Algeria *vs M.m insignitus *from Tunisia lineages	[45]
Geographical barrier: Moulouya River basin, ?	?	the riverine snake *Natrix maura*	3 lineages: Morocco, east Morocco + Algeria, Tunisia+ east Algeria	[46]
MSC?	5	the riverine snake *Natrix maura*	2 lineages: Tunisia *vs *Morocco	[47]
Post MSC	from 5	the salamander *Salamandra algira*	2 lineages: west Morocco *vs *east Morocco + Algeria	[48]
Marine transgression (Edough peninsula)	4.2	the salamander *Salamandra algira*	Morocco *vs *Edough peninsula lineages	[49]
Marine transgression (Edough peninsula)	4.2	the land snail *Turodella sulcata*	*Turodella sulcata *ss *vs Turodella *sp (Enough peninsula)	[50,51]
?	?	the lizard *Acanthodactylus erythrurus belli*	Morocco *vs *Algeria+Tunisia (*A. blanci *) lineages #	[52]
*in situ *differentiation (with Moulouya River basin as possible geographical barrier) post to colonization from Europe	from 3.5	the wall lizard *Podarcis hispanica *s.l.	3 lineages: northen Morocco *vs *southern Morocco *vs *Tunisia	[53,54]
Climatic fluctuations	2.2	the shrew *Crocidura russula*	Moroccan *vs *Tunisian lineages	[55]
	2	the frog *Rana saharica*	2 clades: *R. s. saharica *from Algeria. and *R. s. riodeoroi *from Morocco	[56,57]
?	1.5	the lizard *Psammodromus algirus*	Minor divergence between Morocco *vs *Tunisia clades	[58]
*in situ *differentiation	1.3	the Crested lark *Galerida cristata*	3 lineages: *cristata *(west NA) vs *randonii *(central NA) vs *senegallensis *(east NA)	[59]
Quaternary glacial refuges	1.6-1.0	the wall lizard *Podarcis vaucheri*	8 subsets in Morocco	[60]
Quaternary glacial refuges	LGM (0.018)	the annual grass *Hordeum marinum*	Iberia *vs *Central Mediterranean lineages	[18]
?	?	the gecko *Tarentola mauritanica*	4 lineages: 2 in Morocco. 1 in Algeria. 1 in Tunisa	[61,62]
?	?	the winter pine processionary moth *Thaumetopoea pityocampa/T. wilkinsoni*	Morocco-west Algeria *vs *east Algeria-Tunisia lineages	Kerdelhué, comm. pers.

# more samples in Algeria required for evaluating relationships with *A. blanci *in Tunisia

Finally, Pliocene and Pleistocene climatic fluctuations should have influenced the distribution and the evolution of *C. aspersum *in north Africa. However, signatures left provide insufficient information to infer precisely the modes and timing of the genetic B-C split. The influence of Quaternary climatic changes is more evident in Iberia, where the occurrence of both B and C lineages can be explained by the persistence of several separate refugia throughout the Pleistocene Ice Ages. Recent parallel phylogeographical patterns support the idea that the Iberian Peninsula comprised a "refugia-within-refugia" rather than a single survival area [69]. Amongst the seven putative terrestrial refugia suggested by these authors (Fig 1), the Betic range and Picos de Europa coincide quite strikingly with the tectonic hypothesis proposed and the spatial genetic pattern inferred from allozymes and/or the haplotype distribution in *C. aspersum*. It seems reasonable to assume that each lineage previously differentiated evolved in isolated refugia during glacial periods, such as near the Betic range for the C clade and in the Picos de Europa area for the B lineage. Range expansion during favourable climatic conditions, that is, from the Günz-Mindel stage for B and the Mindel-Riss stage for C, would lead to the contact zone observed in Galicia. Moreover, the occurrence of C haplotypes in the Betic area would agree with the geological scenario based on microplate drift from Oligocene, since the Betic with the Rif Cordillera formed a continuous orogenic belt together with the Kabylies blocks, the Balearic Islands, Calabria, Corsica and Sardinia [31]. However, haplotypes identified in Sardinia and Balearic Islands do not support this scenario but sampling is extremely poor in these areas as in Corsica. For the Tyrrhenian region, populations collected very recently in Sicily but not yet analysed should indicate close relationships with C haplotypes, especially Tunisian ones. Beyond geological assumption of a land connection between Europe and Africa in the continuity of the Maghrebides at the level of the present Sicilian canal until late the Pliocene (2 Myr; [31]), allozyme and morphometric features indicate similarities between southernmost Italian samples analysed and eastern north African populations [9,14]. An alternative assumption of secondary contacts *versus *a differentiation centre in the southern Italian Peninsula could equally lead to resemblance between those peri-Tyrrhenian populations. Although we are very short of sequences from these areas, the derived position from eastern African haplotypes of the unique Italian 16S type would rather favour the hypothesis that Italy would have become a contact zone between African and European populations. Morover, as mentioned above, the most likely north African origin of Mediterranean species supports this scenario.

## Conclusion

While keeping in mind the role of anthropogenic pathways in the spread of the species, time estimates and genetic discontinuities lead to suggest that Pliocene and Pleistocene climatic changes would have played a significant role in shaping variation in north Africa. However, the distribution of haplotypes compared to the present-day position of blocks drifted in the western Mediterranean basin during the Tertiary could not rule out the hypothesis referring to much an older vicariance process to account for the whole biogeographical pattern of *C. aspersum*. Contrary to the tectonic scenario we previously proposed [9], the circum-Mediterranean range of typical eastern types would favour dispersal through plate migration of an eastern ancestor rather than a western one. Subsequent orogenic events such as the Tell Atlas building that may have impeded gene exchange between populations on the two sides of the mountain chain, combined with climatic fluctuations from mid-Pliocene to the last ice age, could thus explain the east-west subdivision in north Africa. The time to the most recent common ancestor estimated for *C. aspersum*, as for each of the B (west) and C (east) lineages, supports this parsimonious scenario. The intermediate position of Kabylia, as regards both geography and genetics, would explain unstable phylogenetic relationships of haplotypes of this area. This main region, recently recognised with Numidia and Kroumiria as a hotspot of biodiversity in the Mediterranean basin [70], would have been first a centre of dispersal and then of secondary contact between isolated lineages (models 1 and 2, Fig 2). The retreat of populations into the Edough Peninsula, that seems to have served as refugia for many species during Pliocene marine transgression, could have accentuated the genetic cleavage between the B and C entities. Subsequent population expansion towards the east when floods ended could partially explain the low nuclear (allozyme) and mitochondrial variability characterizing especially Tunisian samples [9,15]. Transitory and prolonged bottlenecks inherent to successive migrations that likely continued until the LGM would moreover be compatible with demographic expansion observed in that easternmost north African region. Also, northward expansions with climatic improvements since the Mindel-Riss interglacial stage would indicate a loss of the eastern lineage during expansion phases. Whatever the range of *C. aspersum*, the typical eastern populations seem to have been limited in their dispersal after the LGM.

To resume, the different steps of the scenario inferred involve a northward colonization of the western lineage that would have diverged form eastern ancestral type. Most data support the migration model 1, which however needs to be refined in considering the dual role of the Kabylia (Fig 2). In relation to vicariant and dispersal events that could have played a role in the colonization process of both lineages, discrepancies in the success of northward expansion could also reflect variation in adaptive potential of populations among B and C lineages. The extinction probability of populations in changing environments being higher in small than in large populations, one might imagine that typical eastern populations experienced severe and prolonged bottlenecks. As well as demographic studies, efforts should be made to research other possible causes of variation in the colonization process between both lineages. Moreover, this biogeographic scenario should be evaluated (i) by searching for concordant phylogeographic patterns of codistributed species to indicate the influence of common historical factors, (ii) by analysing populations with more extensive sampling, especially in the two putative contact zones identified (Kabylia and Galicia) as in southern Italian Peninsula, and (iii) in incorporating calibration points different from the dubious shell fossils recorded as *C. aspersum*.

## Methods

### Samples

Most individuals analysed here originated from populations previously sampled for anatomical and biochemical surveys [9,12,14,16,71]; other populations were sampled specifically for the current analysis. Overall, 73 localities were considered, 66 and 49 for 16S rRNA and cyt *b *genes respectively (Fig 1). Individuals of the subspecies *maxima *coming from a snail farm in Brittany (France) were also analysed and used as outgroups in phylogenetic and network-based analyses. Sample site characteristics and haplotype codes are given as additional file 1.

### DNA extraction, amplification and sequencing

The protocol followed here has been described in [15]. Briefly, total genomic DNA was obtained from foot and mantle muscles of either fresh or frozen material stored since previous investigations. Protein extracts of liver or muscle were used when foot and mantle were not available. We used either the CTAB, or the chelex extraction protocols for both frozen and fresh material [15]. We amplified fragments of approximately 380 and 560 bp for the 16S rRNA and cyt *b *mitochondrial genes respectively. The 16S fragment was amplified using primers 16S-1 (5'-TGACTGTGCAAAGGTAGC-3') and 16S2 (5'-CTGGCTTACGCCGGTCTG-3') [72]. The cyt *b *region was amplified using Cytb1 (5'-TTATTGAGGCGCTACGGTTAT-3') and Cytb2 (5'-GCAAGCGAAATATAAGGTTCT-3') primers. Amplification of template DNA was carried out in a volume of 25 μl consisting of 10 mM Tris-HCl (ph 9.0), 50 mM KCl, 1.5 mM MgCl_2_, 200 μm of each dNTP, stabilizers including BSA, 1.5 U of Taq DNA polymerase (Ready to Go^®^, Amersham Pharmacia Biotech.), 0.20 μm of each primer and 50 ng of DNA. The PCR conditions were for 16S rRNA, an initial denaturation step of 94°C (5 min), followed by 35 cycles of 94°C (30 s), 50°C (30 s), 72°C (40 s) and a final extension phase at 72°C for 3 min; for cyt *b*, an initial denaturation step of 94°C (5 min), followed by 35 cycles of 94°C (40 s), 50°C (40 s), 72°C (1 min) and a final extension phase at 72°C for 5 min. Double-strand sequences were obtained by direct manual sequencing of the PCR products using the Sequenase v.2 PCR product sequencing kit (Amersham), or using an automated sequencer (PE Applied Biosystems 310 Genetic Analyser, UMR 6553; plate-forme de séquençage et génotypage OUEST-genopole^®^).

### Sequence analysis

Mitochondrial sequences were aligned using CLUSTALW, v1.7 [73]. For the 16S RNA region, they were manually adjusted, based on the secondary structure of the large ribosomal subunit of *C. nemoralis *[74]. The sequences were submitted to GenBank (Additional file 1). Analyses of sequence polymorphism were carried out using DNASP v4.10.9 [75] and ARLEQUIN v3.1 [76].

### Phylogenetic analysis

The degree of phylogenetic signal was first evaluated in both gene data sets by performing the left-skewness (g1) test using 10 000 randomly generated trees under the maximum likelihood criterion, as implemented in PAUP* v4.0b10 [77]. For inferring phylogenetic relationships among individuals, we used maximum likelihood (ML) and bayesian-based inference (BI) methods. The best fit model of nucleotide substitutions was selected prior to ML and BI analyses using the Akaike Information Criterion. The software MrAIC v1.4.2 [78] was used to evaluate the fit of the data to 24 different models of nucleotide substitutions. For both genes, the resulting best fit model was *HKY*, with two different rates for transitions and transversions, unequal base frequencies, a parameter for invariable sites (*I*) and a gamma distribution parameter that describes rate variation across variable sites (Γ). This model of nucleotide substitution was incorporated in PHYML V2.4.4[79] and in MRBAYES v3.1.1-p1 [80] for ML and BI analyses respectively. For ML analysis, the robustness of inferences was assessed by bootstrap resampling using 1000 repetitions. For Bayesian analysis, the posterior probabilities of trees and parameters were approximated with Markov Chain Monte Carlo (MCMC) and Metropolis coupling. For each gene, we ran two independent MCMC analyses with four chains each and a temperature set to 0.2. Each chain was run for 2 000 000 cycles with trees sampled every 100 generations. Posterior probabilities were obtained from the 50% majority rules consensus of trees sampled after discarding the trees saved before chains reached apparent stationarity (i.e. a 'burn-in period" of 50 000 generations for 16S rRNA, 52 000 for cyt *b*). Due to low divergence among individuals and possible persistence of ancestral nodes along with descendants, network-based approaches are better suited for intraspecific phylogeny than tree-based algorithms [81]. Consequently for both mtDNA regions, the median joining algorithm implemented in Network v4.2.0.1 software [82] was also used with default settings for constructing networks (weight = 10 and ε = 0). Due to the high gene diversity in both mtDNA regions, each data set was first reduced using the star contraction option which identifies and contracts any starlike phylogenetic cluster into one ancestral type. Median networks that contained all possible equally short trees were simplified by running the maximum parsimony (MP) calculation option to eliminate superfluous nodes and links.

Before evaluating divergence-time estimates between lineages, we checked whether sequences were evolving at a uniform rate along all branches in a phylogeny. We first performed a molecular-clock likelihood-ratio test (LRT) [83] by comparing the likelihood scores (lnL1 and lnL2) of the same tree constructed under alternative molecular clock assumptions (relaxed *versus *enforced molecular clock). The statistic Δ = 2(lnL1-lnL2) can be compared with a χ^2 ^distribution with (*n*-2) degrees of freedom (where *n *is the number of haplotypes). Secondly we conducted a relative rate test (RRT; [84]) as implemented in RRTree [85] for assessing variation in substitution rates between lineages relative to the outgroup *C. a. maxima*. For the RRT, *p *values were corrected using the Bonferroni method to account for multiple pairwise comparisons when more than two lineages were identified.

### Pattern of migration

Alternate hypotheses of migration were tested by estimating historic migration rates between north Africa and European populations. We compared models employing asymmetric versus symmetric migration rates between European and north African clusters of populations for both genes separately. Because of the small number of European populations in the C (East) haplogroup, estimates were however restricted to the B (West) haplogroup with the Z sequences removed. We used the program MIGRATE-n v. 3.0 [86] to obtain maximum likelihood estimates (MLEs) of theta (θ = *N*μ) and *N*m (effective number of migrants per generation). We obtained MLEs for four models of migration between both regions (bidirectional rates with asymmetric M_NA-E _≠ M_E-NA _or symmetric M_NA-E _= M_E-NA _rates; unidirectional rates with M_NA-E _= 0 or M_E-NA _= 0). We used likelihood ratio tests and the Akaike Information Criterion to choose the model best supported by the data (smaller values of AIC indicating better fit). The program was run with 15 short chains with an increment of 50 and 2000 trees recorded, followed by 3 long chains of 25 000 000 generations each, from which 50 000 trees were sampled after a burnin-period of 5000. We also optimized all models in specifying Ti/Tv ratio (2.56 for cyt *b *and 1.51 for 16S) and gamma distribution shape parameter (α = 0.75 for both genes) from empirical estimates in PAUP* 4.0b [77].

### Demographic analyses

The demographic history of populations was inferred using tests of population growth. Fu's *Fs *[87] and Ramos-Onsins & Rozas' *R*_2 _[88] are among the most powerful statistics to detect demographic expansions; they were estimated using DNASP v4.10.9 [75] and ARLEQUIN v3.1 [76], and their significances were assessed using 1000 coalescent simulated resamplings. We also performed mismatch distribution analyses to evaluate possible historical events of population growth and decline [89]. Indeed, populations that have experienced a rapid expansion in the recent past show unimodal distributions, while those at demographic equilibrium have multimodal distributions. Mismatch distributions were computed for each haplogroup and compared to the expected distributions obtained under a model of sudden expansion.

An approach based on coalescent theory was also used to estimate the demographic history of lineages within *C. aspersum*. Under a coalescent model, it is possible to infer population parameters from genetic sequence data such as mutation rates and divergence time estimates which themselves provide information about effective population sizes through time. We chose the Bayesian Skyline Plot (BSP, [90]) model for inferring past population dynamics. The BSP has the advantage over commonly-used demographic models and skyline plot methods [91] of being independent of a prespecified parametric model of demographic history, and its parameters are directly estimated from sequences, thus limiting the error inherent in phylogenetic reconstruction. The BSP model used MCMC sampling procedures to estimate credibility intervals of the demographic parameters. Parameters were estimated with the software BEAST v1.4.2 [92]. After an optimization step during which parameters of the prior function were changed at each run to reach optimum performance and achieve a reasonable effective sampling size (ESS, number of independent samples of the posterior distribution that the trace is equivalent to) of parameters of interest, we carried out two independent runs of 20 million generations each. Samples of trees and parameters were recorded every 1000 steps. Visualization and diagnosis analysis of each independent BEAST run were done using TRACER v1 [93]. This program leads to redefine the burn-in period (set by default to 10% of the MCMC chain length) and check the convergence of the independent chains. The analysis was repeated after splitting the data into a B *versus *C haplogroup, motivated by earlier results. To provide estimates of time to the most common ancestor (TMCRA) of *C. aspersum *populations, along with the TMCRAs of the B and C lineages (see Results), divergence dates were computed using BEAST. Estimates were based on published mutation rates for land snails. Evolutionary rates at mtDNA genes have been estimated in a few terrestrial gastropods though not in *C. aspersum*. Evidence from Pliocene shell fossils (5.3-1.8 Myr) from several sites around the Mediterranean (Oran, Sicile, Nice; [8]) indicates that *C. aspersum *was already quite widespread in the late Tertiary. However such fossil records, even if shell specimens were assigned to *C. aspersum *species with sufficient confidence, do not allow to go down at the subspecies level (*aspersa or maxima*) even less down to mtDNA lineage within *aspersa*. Information from the fossil record, inadequate for calibrating a molecular clock, can only indicate the minimum age of *C. aspersum*. Estimates in gastropods, intraspecific published estimates vary greatly for 16S rRNA [94-96], as for protein-encoding mitochondrial genes [97-99]. Without more information, we used and tested a range of calibrated rates of change from 0.03 to 5% for cyt *b *excluding the third codon position, and from 0.5 to 10% for 16S rRNA, to calculate a rough estimate of the timing of divergence events. In all cases we used an HKY model of nucleotide substitution. Results were compared with the estimated times based on allozyme evolution (0.08-0.10 DNei/Myr; [32]) which we published previously [9].

## Authors' contributions

This paper continues a series of collaborative studies by the authors on the spatial structure of genetic variation in several land snails. AG's research focuses on the use of molecular approaches mainly in a phylogeographical context. LM is interested in evolutionary ecology using land snails as model species. Both authors read and approved the final manuscript.

## Supplementary Material

Additional file 1 **Geographic location of *C. aspersum *samples and sample size (*n*), name of individuals sequenced and their corresponding cyt *b *and 16S RNA haplotypes, and GenBank accession numbers**. Geographic location of C. aspersum samples analysed in this study (population code correspond to those in Fig 1: A, Algeria; C, Corsica; Ct, Crete; Cr, Croatia; E, Spain; F, France mainland; G, Greece mainland; GB, Great-Britain; I, Italia; M, Morocco; P, Portugal; Sa, Sardinia; T, Tunisia; Tu, Turkey). Sample size (n), name of individuals sequenced (population code and individual number) and their corresponding cyt b and 16S RNA haplotypes and GenBank accession numbers. Two kinds of haplotype codes are provided: (i) the sequence name for unique haplotype, (e.g. A1.1), (ii) ck (k up to 24 distinct types) and sk (k up to 19 distinct types) for cyt b and 16S respectively for haplotypes shared by distinct individuals (*: haplotypes published in [15]). Click here for file
